# Evaluating change in health-related quality of life in adult rhinitis: Responsiveness of the Rhinosinusitis Disability Index

**DOI:** 10.1186/1477-7525-3-68

**Published:** 2005-11-08

**Authors:** Hubert Chen, Patricia P Katz, Stephen Shiboski, Paul D Blanc

**Affiliations:** 1 Department of Medicine, University of California, San Francisco (UCSF), CA, USA; 2 Cardiovascular Research Institute, UCSF, CA, USA; 3 Institute for Health Policy Studies, UCSF, CA, USA; 4 Department of Biostatistics and Epidemiology, UCSF, CA, USA

## Abstract

**Background:**

The Rhinosinusitis Disability Index (RSDI) is a validated measure of health-related quality of life (HRQL) in rhinitis. Responsiveness of the RSDI to changes in health status over time has not been described.

**Methods:**

We studied adults with a self-reported physician diagnosis of rhinitis identified through a national telephone survey. HRQL was assessed at baseline and at 24 months using the RSDI. Symptom severity, physical health status (SF-12 PCS), psychological mood (CES-D), and perceived control of symptoms were also assessed at the time of each interview. In addition, we ascertained specific health outcomes attributed to rhinitis, including days of restricted activity, job effectiveness, number of physician visits, and medication costs.

**Results:**

Of 109 subjects interviewed at baseline, 69 (63%) were re-interviewed 24 months later. RSDI scores improved by = 0.5 standardized response mean in 13 (19%) subjects and worsened in 17 (25%). Change in the RSDI over time correlated with changes in symptom severity (r = 0.38, p = 0.001), physical health (r = -0.39, p = 0.001), mood (r = 0.37, p = 0.002) and perceived control of symptoms (r = -0.37, p = 0.01). In multivariate analyses adjusted for baseline health status, improvement in RSDI was associated with less restricted activity (p = 0.01), increased job effectiveness (p = 0.03), and decreased medication costs (p = 0.05), but was not associated with change in the number of physician visits from baseline (p = 0.45).

**Conclusion:**

The RSDI is responsive to changes in health status and predicts rhinitis-specific health outcomes.

## Background

Rhinitis is a common chronic condition and can be a significant source of impairment. To measure the impact of rhinitis on health-related quality of life (HRQL), several disease-specific measures have been developed [1,2]. These instruments vary in length and content. Certain instruments, such as the 28-item Juniper Rhinoconjunctivitis Quality of Life Questionnaire (RQLQ), were originally intended to focus on persons with allergic disease [3]. Other instruments, such as the 16-item Sino-Nasal Outcome Test (SNOT-16), focus more on conditions characterized by chronic nasal obstruction, typically sinusitis [4].

The Rhinosinusitis Disability Index (RSDI), another measure of HRQL, has been used both in persons with allergic disease as well as in those with chronic nasal obstruction [5,6]. The instrument has 30 items comprising three domains: physical, functional, and emotional. Internal consistency and test-retest reliability of the instrument were initially described in 87 patients with a physician's diagnosis of rhinitis or rhinosinusitis [5]. The RSDI has subsequently been applied in larger populations across a spectrum of rhinologic diagnoses, ranging from allergic rhinitis to chronic sinusitis [6].

In a previous analysis, we evaluated the performance of the RSDI cross-sectionally in 109 adults with predominately allergic disease [7]. We found that the RSDI correlated in the expected ways with other measures of physical and mental health status, providing good evidence of construct validity. Furthermore, we found that psychosocial factors, such as the perception of one's own ability to deal with his or her nasal condition (termed '*perceived control*'), can also play a large role in determining quality of life in rhinitis.

Although the discriminative properties of the RSDI have been studied in a cross-sectional fashion, its responsiveness to changes in health status has not been established. Responsiveness describes the ability of an instrument to capture meaningful changes in health over time. To demonstrate responsiveness, the same instrument must be administered at least twice over a given period of time. Responsiveness, however, differs from test-retest reliability. Test-retest reliability is measured by re-administering the same instrument within a short time interval during which the subject's health status remains unchanged. In contrast, responsiveness is typically demonstrated over longer intervals during which the subject's health status might reasonably have changed in some relevant way. Responsiveness has important implications for instruments used in clinical trials, where investigators are often trying to measure a clinically meaningful change before and after an intervention. In addition, it is also relevant in the study of disease progression or remission.

To determine the responsiveness of the RSDI, we studied change in HRQL in 69 adults with rhinitis using data collected over a 24-month period. Cross-sectional results of the 109 subjects studied at baseline have previously been reported [7]. Responsiveness of the RSDI was tested relative to other health status measures simultaneously administered to assess symptom severity, physical functioning, mood, and perceived control of symptoms. We then used the RSDI to evaluate the relationship between change in HRQL and other rhinitis-specific health outcomes.

## Methods

### Overview

We used data collected as part of a prospective, longitudinal cohort study of adults with asthma and rhinitis. We assessed change in HRQL and health status among 69 subjects with rhinitis alone (without concomitant asthma) who participated in two consecutive interview waves conducted approximately 24 months apart. We evaluated responsiveness of the RSDI relative to other generic and disease-specific health status measures simultaneously assessed. We also evaluated how change in disease-specific HRQL, measured by RSDI, was related to change in other rhinitis-specific health outcomes, including restricted activity, job effectiveness, physician visits, and medication costs.

### Subject selection

Subject data were drawn from a larger prospective study of persons with asthma, rhinitis, or both. Details of recruitment and subsequent interview waves have been previously reported [8]. In brief, we enrolled English- and Spanish-speaking subjects aged 18 to 50 years from the Northern California region via random-digit dialing. Approval for the study of human subjects was obtained from the University of California, San Francisco Committee on Human Research.

We used data from interviews conducted in 2000–1 (baseline for this study) and 2002–3 (follow-up for this study). Subjects were included in our analysis if they reported a physician's diagnosis of allergic rhinitis, sinusitis, hay fever, or chronic post nasal drip, *without *concomitant asthma. One hundred nine subjects completed the rhinitis component of our health survey at baseline, of which 69 (63%) were re-interviewed at follow-up.

### Health status measures

#### Rhinosinusitis Disability Index (RSDI)

To measure disease-specific HRQL in rhinitis, we used the RSDI, a 30-item questionnaire developed for use in persons with nasal or sinus disease [5]. Each item is rated on a 5-point Likert scale ranging from 'never' (scored as 0) to 'always' (scored as 4). The total score possible, calculated by summing the individual items, ranges from 0 to 120, with higher scores reflecting worse HRQL. The RSDI has 3 subscale domains: physical (11 items), functional (9 items), and emotional (10 items). The individual items comprising each of these domains are shown in Additional file: 1. Its reliability and validity have been demonstrated in patients with various rhinologic conditions [6]. In our analysis of subjects at baseline, the RSDI demonstrated high internal consistency with a Cronbach's alpha of 0.97 [7].

#### Rhinitis Symptom Score (RSS-4)

To measure symptom severity in rhinitis, we used four items drawn from a previously validated 31-item symptom scale for rhinoconjunctivitis and asthma developed by Wasserfallen et al [9]. The specific items included in our rhinitis questionnaire assessed four types of symptoms: 'sensation of fullness, congestion or blockage of the nose', 'sneezing', 'sinus headache or pain in face', and 'postnasal drip in back of throat'. Symptoms were rated on a 5-point Likert scale. Scores for the individual items were totaled yielding a possible range of 0 to 16, with higher score reflecting greater symptom severity. Using this 4-item symptom scale in our baseline analysis, we observed good internal consistency with a Cronbach alpha of 0.81.

#### The Short Form 12 (SF-12)

To measure general physical functioning we used the physical component summary (PCS) of the SF-12. The SF-12 consists of 12 items drawn from the widely used Short Form 36 (SF-36). The reliability, validity, and responsiveness of the SF-12 have been demonstrated in various disease states, and are comparable to those of the SF-36 [10]. SF-12 PCS scores range from 0 to 100, with a general population mean of 50 ± 10 (SD). Higher scores reflect better physical functioning.

#### Center for Epidemiologic Studies Depression scale (CES-D)

The CES-D consists of 20 items originally intended to assess depressive symptoms [11]. Further evidence, however, suggests that the CES-D also measures general psychological factors in addition to depression[12,13] For this reason, we refer to the CES-D as assessing 'psychological mood'. CES-D scores range from 0 to 60, with higher scores reflecting worse mood.

#### Perceived Control of Rhinitis Questionnaire (PCRQ)

Psychosocial factors such as an individual's perception of his or her ability to deal with illness, referred to as *perceived control*, have been demonstrated to be an important correlate of health status, both in rhinitis and asthma [7,14], as well as other chronic conditions [15]. To measure perceived control, we used the 8-item PCRQ. PCRQ scores range from 8 to 40, with higher scores reflecting greater perceived control of rhinitis. We previously validated this instrument in a cross-sectional analysis of our baseline data [7]. Similar instruments, upon which it is based, have also been validated for use in asthma [14].

### Disability, health care utilization, and medication costs

Other rhinitis-specific health outcomes assessed included job effectiveness and restricted activity over the 4 weeks prior to interview, and physician visits and medication costs over the 12 months prior to interview. Job effectiveness, in terms of the specific impact of the subject's nasal condition, was assessed on a scale of 0 to 100% (0% meaning unable to work at all and 100% meaning work not affected). Restricted activity was reported as the number of days restricted due to subject's medical condition. Physician visits were reported as the number of visits made specifically for a nasal condition, excluding routine visits for allergy desensitization. Out-of-pocket health costs were assessed separately for prescription and over-the-counter rhinitis medications. Subjects were asked to choose from four possible cost ranges: 'less than $10', '$10 to $99', '$100 to $1,000', or 'more than $1,000'. For the purpose of our analysis, we assigned subjects to the median value for each response category (e.g. $45 for '$10 to $99'), using $1000 for the highest category. A single cost variable was then calculated by summing costs for prescription and over-the-counter medications.

### Statistical analyses

Comparisons between subjects who completed a follow-up interview (n = 69) with those who had not (n = 40) were made using *t *test for continuous variables, Chi-square test for dichotomous variables, and Chi-square test for trend for ordinal variables. Mean (±SD) summary scores were calculated for each health status measure at baseline and 24-month follow-up. Change was calculated by subtracting baseline scores from scores at follow-up and expressed as standardized response means (SRM). The SRM equates to the overall observed change divided by the standard deviation of the individual observed differences in score for the entire group. For the RSDI, a negative change in score reflects improved HRQL over time, whereas a positive change in score reflects worsened HRQL. To evaluate for changes in the RSDI within individuals, we used the paired *t *test.

We examined responsiveness of the RSDI in two separate ways. First, we evaluated change in the RSDI score relative to change in the other health status measures delineated above (RSS-4, SF-12 PCS, CES-D, and PCRQ), treating all variables as continuous. Pearson correlations were used to make these comparisons, as changes in scores approximated normal distributions for all health measures studied. As an alternative analysis, we categorized subjects into three groups, 'better', 'same', or 'worse', based on change in each health status measure. We used a change quantified as one standard error of measurement (SEM) or greater as the criterion for a difference great enough to be consistent with a minimal clinically important difference (MCID). Evidence by Wywrich and others supports use of one SEM (calculated as the standard deviation of the instrument multiplied by the square root of one minus its reliability coefficient) as an approximation of the MCID [16]. By inference, this should also be a reasonable gauge of a substantive difference in score over time. Thus using this categorization scheme, we calculated SEM-based MCIDs for the RSS-4, SF-12 PCS, CES-D, and PCRQ. Based on these cut-offs, we defined subjects as 'same' (absolute change in score <1 SEM), 'better' (score improved by ≥1 SEM), or 'worse' (score worsened by ≥1 SEM) for each measure. Mean change in RSDI was compared among the groups, first using one-way ANOVA to detect an overall difference, then using Tukey's *t *test to perform multiple comparisons (when ANOVA was significant at p < 0.05). We repeated this analysis for each health status measure to which the RSDI was compared (RSS-4, SF-12 PCS, CES-D, and PCRQ).

Finally, we used linear regression to evaluate the relationship between change in HRQL, as measured by the RSDI, and change in rhinitis-specific health outcomes (disability, health care utilization, and medication costs). We first evaluated each health outcome in a bivariate model with change in RSDI score as the independent variable. We then performed multiple linear regression adding baseline RSS-4, SF-12 PCS, CES-D, and PCRQ as covariates in the model, in order to take into account each subject's health status at baseline. Effect estimates are expressed as change in the health outcome variable (days of restricted activity, percent change in job effectiveness, number of visits, or dollars spent for medications) per 1.0 SRM change in the RSDI score. All analyses were performed using SAS System for Windows release 8.02.

## Results

### Subjects characteristics

Overall, the 69 subjects studied were predominately female, white (non-Hispanic), and well-educated, with predominately moderate to severe self-rated rhinitis (Table 1). Baseline characteristics of those subjects re-interviewed were not significantly different that those who were not, with the exception of income. Subjects not re-interviewed were more likely to have an annual family income of less than $40,000.

**Table 1:** Subject characteristics of 109 adults with rhinitis interviewed at baseline

	Re-interviewed at 24 months		
		
Baseline characteristics	Yes (n = 69)	No (n = 40)	p value
Age, mean ± SD	40 ± 8.2	37 ± 8.6	0.16
Female, n (%)	44 (64)	30 (75)	0.23
White (non-Hispanic), n (%)	50 (72)	30 (75)	0.77
Education, n (%)			0.36
High school or less	10 (14)	8 (20)	
Some college	47 (68)	27 (68)	
Graduate degree	12 (17)	5 (13)	
Married, n (%)	44 (64)	23 (58)	0.52
Income <$40,000/y, n (%)	10 (14)	12 (30)	0.05
Smoking status, n (%)			0.46
Never	43 (62)	22 (55)	
Former	15 (22)	10 (25)	
Current	11 (16)	8 (20)	
Self-rated severity of rhinitis, n (%)			0.95
Mild	24 (35)	14 (34)	
Moderate	35 (51)	19 (48)	
Severe	10 (14)	7 (18)	

### Change in HRQL and health status

**Figure 1:**
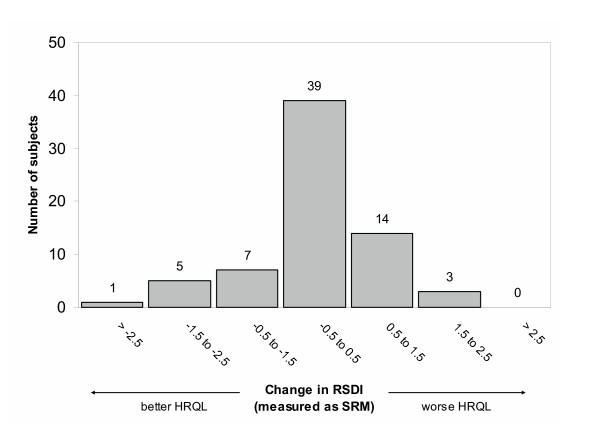
**Change in health-related quality of life (HRQL) over 24 months in 69 adults with rhinitis**. Width of each bar represents one standardized response mean (SRM) calculated as the mean observed change divided by the standard deviation of the observed change. A negative change in RSDI score reflects better HRQL, whereas a positive change in score reflects worse HRQL.

The change in HRQL over follow-up approximated a normal distribution, with the majority of subjects demonstrating a change of less than 0.5 SRM (Figure 1). The RSDI decreased by 0.5 SRM or more (better HRQL) in 13 (19%) subjects and increased by 0.5 SRM or more (worse HRQL) in 17 (25%). Overall, RSDI scores for the group as a whole did not change significantly (Table 2). Similarly, no statistically significant differences were observed in any of the RSDI subscales.

**Table 2:** Change in health-related quality of life and health status among 69 adults with rhinitis

Measure	Baseline (mean ± SD)	24 month follow-up (mean ± SD)	Observed change* (mean ± SE)	Standardized response mean^†^	p value^‡^
RSDI – Total	23.3 ± 23.4	24.6 ± 24.0	1.3 ± 2.7	0.06	0.63
*Physical*	11.2 ± 10.6	10.7 ± 9.6	-0.4 ± 1.2	-0.04	0.72
*Functional*	5.6 ± 7.0	6.5 ± 7.5	0.9 ± 0.9	0.13	0.28
*Emotional*	6.6 ± 7.7	7.3 ± 8.4	0.8 ± 1.0	0.10	0.42
RSS-4	6.9 ± 4.6	5.4 ± 3.9	-1.5 ± 0.5	-0.31	0.01
CES-D	10.5 ± 10	11.5 ± 9.9	1.0 ± 1.3	0.09	0.43
SF-12 PCS	48.2 ± 8.6	47.8 ± 9.0	-0.5 ± 1.0	-0.05	0.65
PCRQ	27.7 ± 4.8	28.8 ± 5.2	1.2 ± 0.7	0.21	0.09

RSDI = Rhinosinusitis Disability Index; RSS-4 = 4-item Rhinitis Symptom Score; CES-D = Center for Epidemiologic Studies Depression scale; SF-12 PCS = Short Form 12 Physical Component Summary; PCRQ = Perceived Control of Rhinitis Questionnaire. * Observed change = (score at follow-up) - (score at baseline). † Standardized response mean = [(score at follow-up) - (score at baseline)/(SD of observed change)]. ‡ Paired *t *test

Changes in symptom severity, physical functioning, mood, and perceived control also approximated normal distributions. Rhinitis symptom severity, as measured by the RSS-4, improved by a small, though significant, increment (Table 2). The remaining health status measures (SF-12 PCS, CES-D, and PCRQ) demonstrated no statistically significant changes at the group level.

### Responsiveness to changes in health status

Change in the RSDI at the individual level correlated moderately well with changes in the RSS-4, SF-12 PCS, CES-D, and PCRQ. Moreover, changes were in the anticipated directions (Table 3). Specifically, better HRQL, as measured by the RSDI, was associated with lower symptom severity, greater physical functioning, better mood, and greater perceived control. Similar correlations were observed for each of the RSDI subscales.

**Table 3:** Correlations between change in the RSDI and change in other health status measures

	Δ RSS-4	Δ SF-12 PCS	Δ CES-D	Δ PCRQ
Δ RSDI – Total	0.38 (0.16, 0.57)	-0.39 (-0.57, -0.16)	0.37 (0.15, 0.56)	-0.37 (-0.56, -0.14)
*Physical*	0.32 (0.09, 0.52)	-0.37 (-0.55, -0.14)	0.31 (0.08, 0.51)	-0.30 (-0.50, -0.07)
*Functional*	0.39 (0.17, 0.58)	-0.45 (-0.62, -0.23)	0.39 (0.17, 0.57)	-0.39 (-0.57, -0.17)
*Emotional*	0.33 (0.10, 0.52)	-0.23 (-0.44, 0.01)	0.31 (0.08, 0.51)	-0.32 (-0.51, -0.09)

Values represent Pearson correlation coefficients (95% confidence interval). RSDI = Rhinosinusitis Disability Index; RSS-4 = 4-item Rhinitis Symptom Score; CES-D = Center for Epidemiologic Studies Depression scale; SF-12 PCS = Short Form 12 Physical Component Summary; PCRQ = Perceived Control of Rhinitis Questionnaire. Δ = Change over 24 month follow-up.

**Figure 2:**
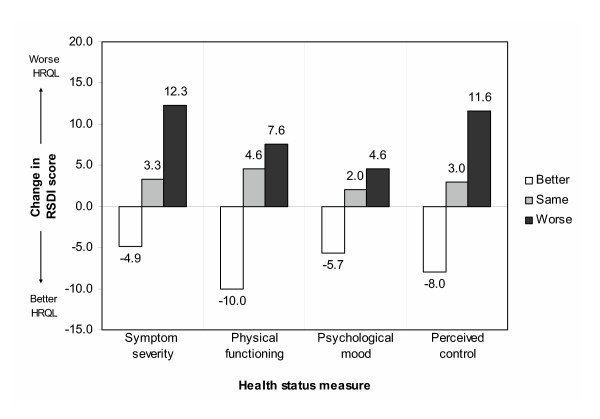
**Responsiveness of RSDI to change in other health status measures**. Bars represent mean change in RSDI score categorized by change in health status (better/same/worse, see legend above). A negative bars (decrease in RDSI) reflect better health-related quality of life (HRQL), whereas a positive bars (increase in RSDI) reflect worse HRQL. Symptom severity, physical functioning, psychological mood, and perceived control of rhinitis were assessed using the RSS-4, SF-12 PCS, CES-D, and PCRQ, respectively (see Methods).

We reanalyzed these relationships, categorizing subjects based on change in health status as measured by the RSS-4, SF-12 PCS, CES-D, and PCRQ. Mean RSDI scores improved (negative change in score) in subjects categorized as 'better' with respect to symptom severity (RSS-4), physical functioning (SF-12 PCS), mood (CES-D), and perceived control (PCRQ) (Figure 2). Similarly, mean RSDI scores worsened (positive change in score) in subjects categorized as 'worse' according to the other health status measures. In all cases, observed changes in the RSDI were statistically significant compared to subjects categorized as the 'same' relative to baseline.

**Figure 3:**
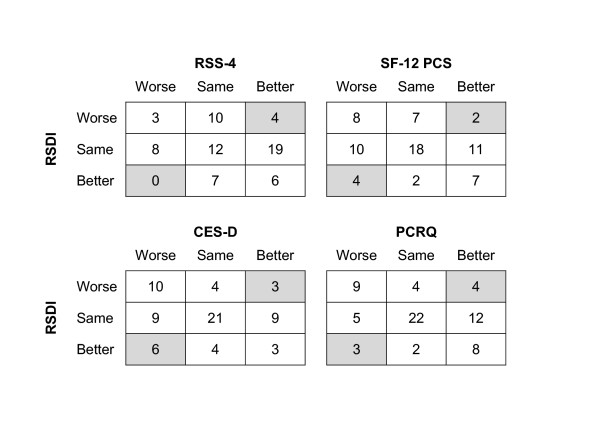
**Frequency counts for categorical change between the RSDI and other health status measures**. Values represent the number of subjects within each category. Shaded cells indicate highly discordant change between measures. Overall, such discordance occurred in approximately one in every 10 subjects. Change in the RSDI was highly discordant in 6% of subjects for the RSS-4, 9% of subjects of the SF-12 PCS, 13% of subjects for the CES-D, and 10% of subjects for the PCRQ. For definitions of abbreviations see Table 2.

These same results are presented again in an alternative format in Figure 3, showing categorical change in the RSDI versus categorical change in the other health status measures. Highly discordant change between measures (for example, 'better' versus 'worse') was observed in approximately one in every 10 subjects: 4 (6%) subjects for the symptom severity (RSS-4), 6 (9%) subjects for physical functioning (SF-12 PCS), 9 (13%) subjects for mood (CES-D), and 7 (10%) subjects for perceived control (PCRQ).

### Relationship with self-reported health outcomes

Change in HRQL, as measured by the RSDI, was significantly associated with change in days of restricted activity, altered job effectiveness, and incremental medication costs, but was not associated with differing frequency of physician visits relative to baseline (Table 4). After adjusting for baseline health status (RSS-4, SF-12 PCS, CES-D, and PCRQ), these observed associations for activities, effectiveness and medication costs remained statistically significant. In these multivariate analyses, an increase in RSDI score of 1.0 SRM (22.3 points) was associated with an increase of nearly 2.5 days of restricted activity per month, a decrease of >4% in job effectiveness, and an increase of >$78 spent on rhinitis medications per year.

**Table 4:** Relationship between change in RSDI and change in rhinitis-specific health outcomes

		Change in RSDI as a predictor of change in health outcome					
		
		Unadjusted model			Adjusted model		
			
Dependent variable	Mean change ± SD	β	± SE	p value	β	± SE	p value
Restricted activity (days per month)	2.4 ± 7.3	2.48	± 0.84	<0.01	2.48	± 0.98	0.01
Job effectiveness* (% effectiveness)	-2.4 ± 12.8	-4.78	± 2.03	0.02	-4.27	± 1.95	0.03
Physician visits (# of visits per yr)	-0.1 ± 3.1	0.41	± 0.46	0.38	0.36	± 0.47	0.45
Cost of medications ($ per year)	4.9 ± 309	87.44	± 36.16	0.02	78.52	± 38.73	0.05

Effect estimates are reported per 1.0 SRM change in the RSDI score. Adjusted model includes baseline symptom severity (RSS-4), physical functioning (SF-12 PCS), psychological distress (CES-D), and perceived control of disease (PCRQ). * Data not available for 24 subjects (9 unemployed, 11 house keeping, 2 attending school, 1 retired, 1 other).

## Discussion

In this study, we demonstrate that the RSDI is responsive to changes in health status over time, and thus can be used to measure longitudinal change in HRQL in rhinitis. Change in HRQL, as measured by the RSDI, correlated in the expected ways with changes in symptom severity, physical functioning, mood, and perceived control of symptoms. In addition, we found that change in HRQL was associated with changes in rhinitis-specific health outcomes, specifically days of restricted activity, job effectiveness, and medication costs, even after controlling for health status at baseline.

Although several measures of HRQL have been developed for rhinitis and sinusitis, few have undergone rigorous psychometric testing [2]. In order for an instrument to be useful, it should be valid, reliable, and responsive. A systematic review by Linder et al. identified 16 instruments used to measure HRQL in sinusitis, of which only three instruments met basic requirements for validity, reliability, and responsiveness: the Chronic Sinusitis Survey – Duration-based (CSS-D), the Rhinosinusitis Outcome Measure-31 (ROM-31), and the SNOT-16 [17]. Additionally, the Juniper RQLQ has also been shown to demonstrate strong measurement properties, particularly in patients with allergic rhinitis [18].

This study focuses on demonstrating the responsiveness of the RSDI, which has yet to be reported. We believe that this is important because the RSDI is useful across a range of rhinologic conditions, is reasonably short in length, and is structured in a way that facilitates telephone administration. These attributes that make the RSDI particularly suitable for repeated administration in longitudinal survey research where the ability to measure change is often desired.

Responsiveness can be determined in a number of ways, but must include some type of longitudinal assessment [19]. There are two general approaches to interpreting change in HRQL, a 'distribution-based' approach (also referred to as 'internal responsiveness') and an 'anchor-based' approach (also referred to as 'external responsiveness') [20,21].

The 'distribution-based' approach relies entirely on the statistical distribution of the results, using effect size or SRM as a method for assessing change. For our initial analysis, we adopted this approach to summarize global changes in the RSDI, expressing the difference in scores as standardized response means (Figure 1), and testing the difference using paired *t *test (Table 2). The main criticism of relying solely on a 'distribution-based' approach is that there is no standard by which to judge whether the changes observed are in fact important to the patient or clinically meaningful. Often this approach is used in clinical trials involving an intervention that is believed to be efficacious. The RSDI has only been used in two clinical trials, neither of which purported to directly assess the responsiveness of the instrument. In one trial evaluating the efficacy of hypertonic saline nasal irrigation, RSDI scores improved by 6.0 to 15.5 points in the treated group compared with controls [22]. In our analysis, we used 0.5 SRM as a measure of change, which corresponds to approximately 11 points on the RSDI. Another trial, which evaluated the utility of a treatment protocol for rhinosinusitis, showed no statistical difference in RSDI scores between those who felt improved and those who did not, although there were measurable differences in other clinical outcomes [23].

Unlike the 'distribution-based' approach, 'anchor-based' approaches attempt to compare, or anchor, changes in a measure to some external criterion. This can be particularly troublesome for HRQL instruments, where no gold standard exists. In this case, the instrument must be compared to other established measures that assess related constructs. Cross-sectional studies in rhinitis have demonstrated that objective clinical measures, such as CT scoring and nasal endoscopy, correlate poorly with symptom severity and quality of life [24,25]. Therefore, it is difficult to argue that responsiveness should be measured in these terms. Instead, we chose to measure the responsiveness of the RSDI relative to other related health status measures to which it has already been shown to correlate with cross-sectionally.

In a previous analysis, we demonstrated that HRQL in rhinitis, as measured by the RSDI, correlates with measures of symptom severity, physical functioning, mood, and perceived control assessed simultaneously [7]. In this current analysis, we studied a subset of these subjects for whom longitudinal data was available. Because subjects were studied 2 years after baseline, enough time had elapsed for health status to have improved in some (either spontaneously or due to medical intervention) and to have worsened in others (due the disease progression or under-treatment). We measured responsiveness of the RSDI to these changes in health status in two ways. We did this first, by correlating change in the RSDI with change in other health status measures on a continuous scale (Table 3) and second, by dividing subjects into categories (better, same, worse) based on significant changes in health status and comparing differences in RSDI scores between categories (Figure 2). Although these analyses may be statistically similar, they are conceptually different. Using both of these methods, we found that the RSDI performed in the hypothesized manner. Additionally, we also compared categorical change in the RSDI versus categorical change in the other health status measures (Figure 3). Presented in this way, we found extreme discordance between measures in only a minority of cases, also consistent with an overall responsiveness to change.

Finally, to provide further support for utility of the RSDI as a measure of change, we used the RSDI to evaluate the relationship between change in HRQL and change in specific health outcomes that we believed to be most relevant to patients suffering from rhinitis. We found that, even after controlling for baseline health status, change in the RSDI score was indeed an independent predictor of change in days of restricted activity, job effectiveness, and medication costs.

We failed to demonstrate, however, that the RSDI is responsive to change in number of physicians visits. This negative finding could be explained by a number of reasons. First, there was little change in the number of visits observed for the group as a whole. Second, physician visits were queried over the 12 months prior to interview as opposed to the RSDI, which was has a recall period of only 4 weeks. Finally, patients with rhinitis often self-manage their own symptoms, and therefore fluctuations in disease severity may not be necessarily be captured by physician visits.

Due to the lack of consensus on the single, best method for determining responsiveness [19], we used a combination of approaches to try to assess whether the RSDI is sensitive to meaningful changes in other measures that should correlate with HRQL. We recognize that the strength of our conclusions can only be as strong as the health status measures we have chosen to use for comparison. This limitation, however, is inherent to any study which proposes to measure the responsiveness of HRQL instruments, given that no gold standard exists.

Some might contend that because health status did not change for the study population as a whole over the 2 years, then what we are in fact measuring is longitudinal construct validity, rather than true responsiveness. This particular distinction remains a topic of ongoing debate [19]. Certain authors have argued that responsiveness represents a psychometric property separate from validity [26,27], whereas others believe that responsiveness should be treated as a form of longitudinal validity [28,29]. When dealing with the measurement of HRQL, this distinction becomes even less clear. Bearing these limitations in mind, the longitudinal characteristics of the RSDI demonstrated in this study meet and exceed most performance expectations for an evaluative HRQL instrument.

## Conclusion

In summary, we conclude that RSDI is responsive to changes in HRQL as indicated by its correlation with other health status measures and rhinitis-specific outcomes measured longitudinally. Because the treatment for rhinosinusitis is based primarily on symptoms and their impact on the individual, it is important to have quantifiable measures that are sensitive to change in health status. Based on the responsiveness of the RSDI we observed, combined with its ease of administration and applicability, this instrument should be considered for future use in other clinical studies of rhinitis and sinusitis.

## List of abbreviations

CCS-D: Chronic Sinusitis Survey – Duration-based

CES-D: Center for Epidemiologic Studies depression scale

HRQL: Health-related quality of life

MCID: Minimal clinically important difference

PCRQ: Perceived Control of Rhinitis Questionnaire

ROM-31: 31-item Rhinosinusitis Outcome Measure

RQLQ: Rhinoconjunctivitis Quality of Life Questionnaire

RSDI: Rhinosinusitis Disability Index

RSS-4: 4-item Rhinitis Symptom Score

SEM: Standard error of measurement

SF-12 PCS: Short Form 12 physical component summary

SNOT-16: 16-item Sino-Nasal Outcome Test

SRM: Standardized response mean

## Authors' contributions

HC conceived and designed the study, performed the statistical analysis, and drafted the manuscript. PB contributed to conception and design of the study, provided the data on which this analysis was based, participated in the interpretation of data, and made substantial revisions to the manuscript. PK provided expertise on psychometric analysis, participated in the interpretation of data, and critically reviewed the final manuscript. SS provided statistical consultation and critically reviewed the final manuscript.

## Supplementary Material

Additional file 1 The Rhinosinusitis Disability Index (RSDI) Domains and Items. Click here for file
