# Supplementary material for: Evaluating change in health-related quality of life in adult rhinitis: Responsiveness of the Rhinosinusitis Disability Index
Source: Health Qual Life Outcomes. 2005 Nov 8;3:68. doi: 10.1186/1477-7525-3-68 (PMC1308842; doi:10.1186/1477-7525-3-68)
Supplement: Additional file 1 — The Rhinosinusitis Disability Index (RSDI) Domains and Items. [file 1477-7525-3-68-S1.doc]

**Appendix: The Rhinosinusitis Disability Index (RSDI) Domains and Items**

| **Physical** (11 items) |
| --- |
| 1. The pain or pressure in my face makes it difficult for me to concentrate 2. The pain in my eyes makes it difficult for me to read 3. I have difficulty stooping over to lift objects because of face pressure 4. Because of my problem I have difficulty with strenuous yard work and housework 5. Straining increases or worsens my problem 6. I am inconvenienced by my chronic runny nose 7. Food does not taste good because of my change in smell 8. My frequent sniffing is irritating to my friends and family 9. Because of my problem I don’t sleep well 10. I have difficulty with exertion due to my nasal obstruction 11. My sexual activity is affected by my problem |
| **Functional** (9 items) |
| 1. Because of my problem I feel handicapped 2. Because of my problem I feel restricted in performance of my routine daily activities 3. Because of my problem I restrict my recreational activities 4. Because of my problem I feel frustrated 5. Because of my problem I feel fatigued 6. Because of my problem I avoid traveling 7. Because of my problem I miss work or social activities 8. My outlook on the world is affected by my problem 9. Because of my problem I find it difficult to focus my attention away from my problem and on other things |
| **Emotional** (10 items) |
| 1. Because of my problem I feel stressed in relationships with friends and family 2. Because of my problem I feel confused 3. Because of my problem I have difficulty paying attention 4. Because of my problem I avoid being around people 5. Because of my problem I am frequently angry 6. Because of my problem I do not like to socialize 7. Because of my problem I frequently feel tense 8. Because of my problem I frequently feel irritable 9. Because of my problem I am depressed 10. My problem places stress on my relationships with members of my family or friends |
|  |
